# Fission Yeast Tel1^ATM^ and Rad3^ATR^ Promote Telomere Protection and Telomerase Recruitment

**DOI:** 10.1371/journal.pgen.1000622

**Published:** 2009-08-28

**Authors:** Bettina A. Moser, Lakxmi Subramanian, Lyne Khair, Ya-Ting Chang, Toru M. Nakamura

**Affiliations:** 1 Department of Biochemistry and Molecular Genetics, University of Illinois at Chicago, Chicago, Illinois, United States of America; University of Washington, United States of America

## Abstract

The checkpoint kinases ATM and ATR are redundantly required for maintenance of stable telomeres in diverse organisms, including budding and fission yeasts, *Arabidopsis*, *Drosophila*, and mammals. However, the molecular basis for telomere instability in cells lacking ATM and ATR has not yet been elucidated fully in organisms that utilize both the telomere protection complex shelterin and telomerase to maintain telomeres, such as fission yeast and humans. Here, we demonstrate by quantitative chromatin immunoprecipitation (ChIP) assays that simultaneous loss of Tel1^ATM^ and Rad3^ATR^ kinases leads to a defect in recruitment of telomerase to telomeres, reduced binding of the shelterin complex subunits Ccq1 and Tpz1, and increased binding of RPA and homologous recombination repair factors to telomeres. Moreover, we show that interaction between Tpz1-Ccq1 and telomerase, thought to be important for telomerase recruitment to telomeres, is disrupted in *tel1Δ rad3Δ* cells. Thus, Tel1^ATM^ and Rad3^ATR^ are redundantly required for both protection of telomeres against recombination and promotion of telomerase recruitment. Based on our current findings, we propose the existence of a regulatory loop between Tel1^ATM^/Rad3^ATR^ kinases and Tpz1-Ccq1 to ensure proper protection and maintenance of telomeres in fission yeast.

## Author Summary

Stable maintenance of telomeres is critical to preserve genomic integrity and to prevent accumulation of undesired mutations that might lead to formation of tumor cells. Fission yeast cells serve as a particularly attractive model system to study telomere maintenance mechanisms, since proteins critical for telomere maintenance are highly conserved between fission yeast and humans. Previous studies have shown that the checkpoint kinases ATM (Tel1) and ATR (Rad3) are required for stable maintenance of telomeres in a wide variety of organisms. Here, we investigated the molecular basis for telomere dysfunction in fission yeast cells lacking ATM and ATR kinases. Our results show that fission yeast ATM and ATR are redundantly required to promote efficient recruitment of telomere protection complex subunits to telomeres, which in turn promote recruitment of telomerase needed to maintain telomeres. Human ATM and ATR kinases might similarly promote telomere protection and telomerase recruitment by promoting recruitment of telomere protection complex subunits.

## Introduction

Telomeres, the nucleoprotein protective structures at ends of eukaryotic chromosomes, are essential for stable maintenance of eukaryotic genomes [1]. In most eukaryotic species, telomeric DNA is made up of short repetitive G-rich sequences that can be extended by the specialized reverse transcriptase telomerase, to overcome the inability of semi-conservative DNA replication machineries to fully replicate ends of linear DNA [2]. While most of the telomeric G-rich repeats are composed of double-stranded DNA (dsDNA), telomeres end with G-rich 3′ single-stranded DNA (ssDNA), known as G-tail. Both dsDNA and ssDNA portions are important for maintaining functional telomeres as they provide binding sites for telomeric repeat sequence-specific binding proteins, as well as various DNA repair and checkpoint proteins, that are critical for proper maintenance of telomeres.

In mammalian cells, the shelterin complex, composed of TRF1, TRF2, TIN2, RAP1, TPP1 and POT1, plays critical roles in the stable maintenance of telomeres [1]. TRF1 and TRF2 bind specifically to telomeric dsDNA G-rich repeats via their C-terminal myb-like DNA binding domain, while POT1 binds to the telomeric G-tail via its N-terminal OB-fold domains [1]. On the other hand, RAP1, despite the fact that it is evolutionarily related to the budding yeast dsDNA telomeric repeat-binding protein Rap1, cannot directly bind to DNA, and it is recruited to telomeres via its interaction with TRF2 [1]. Likewise, TIN2 is recruited to telomeres by its ability to interact with both TRF1 and TRF2 [3]. TIN2 plays a central role in the formation of the shelterin complex through its ability to interact with the POT1 binding partner TPP1. Previous studies have shown that TRF2 is essential for preventing fusion of telomeres by non-homologous end-joining (NHEJ) and for attenuating ATM-dependent checkpoint signaling [4]. On the other hand, POT1 is critical for protection of telomeres against nucleolytic processing and for attenuating ATR-dependent checkpoint signaling [4]. The POT1-TPP1 sub-complex was also found to interact with the telomerase complex and to increase processivity of telomerase [5],[6].

Fission yeast *Schizosaccharomyces pombe* is an attractive model system for understanding how the shelterin complex contributes to telomere function since this organism utilizes proteins that show a high degree of conservation to the mammalian shelterin subunits [7]. In contrast, the more extensively studied budding yeast *Saccharomyces cerevisiae*, while providing unparalleled detailed molecular understanding on how telomere maintenance is regulated, cannot provide much insight into how the shelterin components might contribute to telomere function, since budding yeast lacks shelterin and relies on evolutionarily unrelated alternative protein complexes to maintain telomeres [8],[9].

The *S. pombe* shelterin complex is composed of Taz1, Rap1, Poz1, Ccq1, Tpz1 and Pot1 [7]. Taz1 directly binds to telomeric dsDNA G-rich repeats via its myb DNA-binding domain, and is thought to fulfill functions analogous to mammalian TRF1 and TRF2 [10]. Rap1, like mammalian Rap1, does not bind directly to telomeric DNA, but it is recruited to telomeres through its interaction with Taz1 [11],[12]. Poz1, the functional counterpart of mammalian TIN2, connects Taz1 to the G-tail binding protein Pot1 by simultaneously interacting with Rap1 and the Pot1 interaction partner Tpz1 [7]. Deletion of *taz1^+^*, *rap1^+^* or *poz1^+^* causes massive telomerase-dependent expansion of the G-rich repeat-tract length, and thus they are implicated in the negative regulation of telomerase activity [7],[13]. Tpz1, an ortholog of mammalian TPP1, interacts with Pot1 via its N-terminus, and with Poz1 and Ccq1 via its C-terminus [7]. Thus, Tpz1 is the central protein necessary for the formation of the Pot1 sub-complex, composed of Pot1, Tpz1, Ccq1 and Poz1. Pot1 and Tpz1 are both essential for protecting telomeres in fission yeast since deletion of *pot1^+^* or *tpz1^+^* results in rapid and complete loss of telomeric G-rich repeats and chromosome circularization [7].

Ccq1 is required for telomerase-dependent telomere maintenance as well as inhibition of checkpoint responses and recombination at telomeres [7],[14]. While an ortholog of Ccq1 has not been identified in mammalian cells, analogous proteins that are critical for telomerase recruitment and inhibition of checkpoint and repair responses at telomeres might await discovery in mammalian cells. The telomere protection function fulfilled by Pot1 and Tpz1 appears to be provided redundantly by Poz1 and Ccq1, since *poz1Δ ccq1Δ* cells, but not *poz1Δ* or *ccq1Δ* single deletion cells, rapidly lose telomeres and circularize chromosomes [7].

Similar to *pot1Δ* or *tpz1Δ* cells, *S. pombe* cells deleted for either Stn1 or Ten1 rapidly lose telomeres and circularize chromosomes [15]. Fission yeast Stn1 and Ten1 are evolutionarily conserved to *S. cerevisiae* Stn1 and Ten1, which are essential for telomere capping in budding yeast. Budding yeast Stn1 and Ten1 form a complex with the telomeric G-tail binding protein Cdc13, and the Cdc13-Stn1-Ten1 complex has been proposed to represent a telomere-specific replication protein A (RPA)-like complex [16]. Since Pot1 does not appear to be in the same complex as Stn1 and Ten1, fission yeast cells seem to utilize two independent capping complexes to protect telomeres [15],[17]. Higher eukaryotic cells may also utilize both Pot1 and Stn1 complexes to protect telomeres since the Stn1 ortholog in *Arabidopsis* was found to be important for telomere protection, and potential Stn1 orthologs have been identified in mammalian genomes based on sequence analysis [15],[16],[18].

Telomere proteins, such as TRF2 and POT1, inhibit DNA damage and/or DNA replication checkpoint signaling regulated by ATM and ATR kinases [4]. Paradoxically, checkpoint and DNA repair proteins are also essential for stable telomere maintenance. In fact, cells simultaneously lacking both ATM and ATR pathways suffer severe telomere dysfunction in a wide variety of organisms, including budding and fission yeasts, *Arabidopsis* and *Drosophila* [19]–[23]. In budding yeast, where the shelterin complex is absent, studies have uncovered redundant roles for Tel1^ATM^ and Mec1^ATR^ in promoting telomerase recruitment via phosphorylation of Cdc13 to enhance the interaction between Cdc13 and the Est1 subunit of telomerase [24]. However, no molecular details of telomere defect(s) caused by simultaneous loss of ATM and ATR pathways were available for the organisms that utilize telomerase, shelterin, and the Stn1 complex to maintain telomeres. Therefore, we utilized fission yeast to define the nature of telomere dysfunction in cells lacking both Tel1^ATM^ and Rad3^ATR^. Our analyses implicate a defect in efficient accumulation of the shelterin complex subunits Tpz1 and Ccq1 to telomeres as the main cause of telomere dysfunction in *tel1Δ rad3Δ* cells, which exhibit defects in both telomere protection and telomerase recruitment.

## Results

### *tel1Δ rad3Δ* Cells Are Defective in Telomere Protection

In budding yeast, a telomere maintenance defect observed in *tel1Δ mec1Δ* double mutant cells can be suppressed by deleting Rif1 or Rif2 (Rap1 interacting factors) or by reducing Rap1 accumulation at telomeres. These observations suggested that the requirement of Tel1^ATM^ and Mec1^ATR^ for telomere maintenance could be bypassed simply by making telomeres more accessible to telomerase by removing inhibitory regulators of telomerase [25]. Moreover, *tel1Δ mec1Δ* cells lost their viability slower than telomerase RNA mutant (*tlc1Δ*) cells, and *tel1Δ mec1Δ tlc1Δ* cells lost their viability with a rate comparable to *tlc1Δ* cells. Thus, the telomere maintenance defect observed in *tel1Δ mec1Δ* cells may entirely be attributable to the failure of the double mutant cells to efficiently recruit telomerase to telomeres [25].

In contrast, our previous analyses suggested that fission yeast lacking Tel1^ATM^ and Rad3^ATR^ are likely to be defective in telomerase recruitment and other additional functions such as telomere protection [26]. This prediction was made based on the following observations. First, *tel1Δ rad3Δ* cells lost their viability faster than telomerase mutant (*trt1Δ*) cells. Second, *tel1Δ rad3Δ trt1Δ* and *tel1Δ rad3Δ* cells lost their viability at comparable rates, suggesting telomere defects observed in *tel1Δ rad3Δ* cells include a defect in telomerase function. Third, Taz1 deletion (*taz1Δ*), which allows *trt1Δ* cells to stably maintain telomeres by recombination and thus should be able to suppress chromosome circularization if telomerase recruitment is the only defect caused by *tel1Δ rad3Δ*, could not suppress chromosome circularization of *tel1Δ rad3Δ* cells [26],[27].

**Figure 1:**
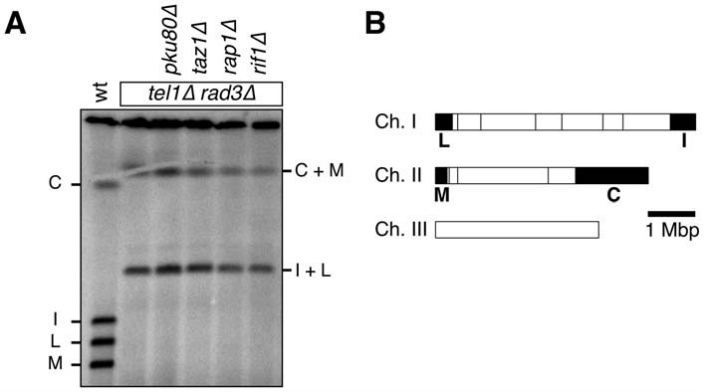
Elimination of Ku80, Taz1, Rap1, or Rif1 cannot suppress chromosome circularization observed in *tel1Δ rad3Δ* cells. (A) Chromosomal DNA from indicated strains was prepared in agarose plugs after cells were extensively restreaked on agar plates. NotI-digested DNA was then used for pulsed-field gel electrophoresis, transferred to Nylon membrane, and hybridized to probes specific for telomeric C, I, L, and M fragments [47]. (B) A NotI restriction map of *S. pombe* chromosomes, shown with telomeric C, I, L, and M fragments marked as black boxes.

However, since *taz1Δ* cells show more severe telomere defects than *rap1Δ* or *rif1Δ* cells [13],[28], we tested if *rap1Δ* or *rif1Δ* could suppress chromosome circularization of *tel1Δ rad3Δ* cells. Fission yeast Rap1 and Rif1 show sequence homology to budding yeast Rap1 and Rif1, respectively, and *rap1Δ* and *rif1Δ* cells carry elongated telomeres, suggesting that they are important for negative regulation of telomerase in fission yeast [11]. However, neither *rap1Δ* nor *rif1Δ* was able to suppress the chromosome circularization phenotype of *tel1Δ rad3Δ* cells (Figure 1A). These results thus establish that mutations of telomerase inhibitors cannot suppress the telomere maintenance defect of *tel1Δ rad3Δ*, and further support the notion that Tel1^ATM^ and Rad3^ATR^ may contribute to telomere protection.

### Tel1 and Rad3 Are Required To Prevent G-Tail Elongation and Accumulation of RPA and DNA Repair Factors at Telomeres

**Figure 2:**
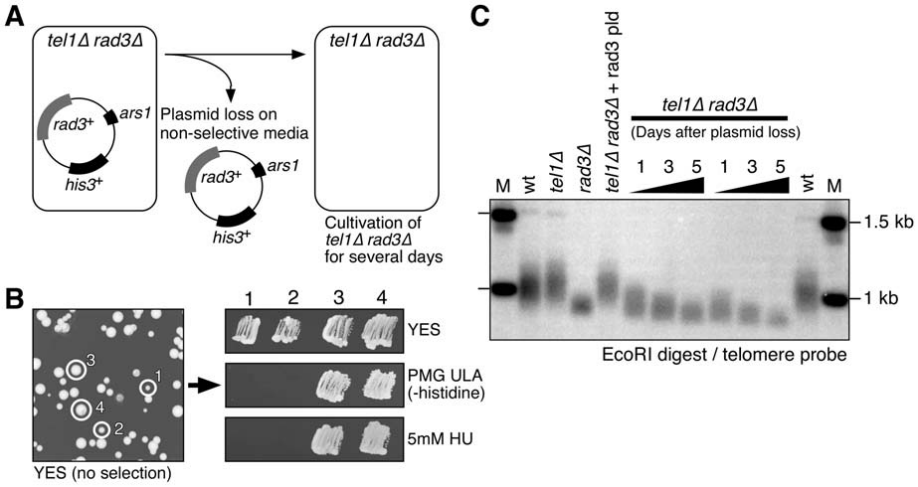
A Rad3-plasmid loss system developed to study telomere dysfunction in *tel1Δ rad3Δ* cells. (A) Experimental scheme for the Rad3-plasmid loss system. (B) Examples of colonies that have lost (1 & 2) or retained (3 & 4) the Rad3-plasmid on YES plate. Colonies that have lost the Rad3-plasmid can be easily identified by their smaller colony size compared to those that retained the plasmid. The absence of the Rad3-plasmid can be further confirmed by the sensitivity of cells to the DNA replication inhibitor hydroxyurea (HU) and their inability to grow on plates lacking histidine. (C) Telomere length analysis for *tel1Δ rad3Δ* cells after loss of the Rad3-plasmid. Genomic DNA samples from indicated cells were prepared, digested with EcoRI, and processed for Southern blot analyses using a telomere repeat-specific probe. Cells used for experiments throughout this paper are equivalent to cells at day 1–2 after plasmid loss.

Next, we tested more directly if loss of Tel1^ATM^ and Rad3^ATR^ causes defects in telomere protection. In order to reliably examine changes in telomere structure or recruitment of various telomere-associated factors in *tel1Δ rad3Δ* cells prior to chromosome circularization, we first developed a new plasmid-based system that allowed us to utilize younger generation *tel1Δ rad3Δ* cells for our experiments, rather than performing meiotic crosses to create *tel1Δ rad3Δ* cells (Figure 2A). In this system, we took advantage of the fact that *tel1Δ rad3Δ* cells carrying a Rad3-plasmid grow significantly slower upon loss of the plasmid, and thus form smaller colonies when grown on non-selective media plates (Figure 2B). For our experiments, we chose multiple small colonies, individually confirmed to be *tel1Δ rad3Δ* based on their inability to grow on media lacking histidine (loss of *his3^+^* marker) or media containing hydroxyurea (loss of *rad3^+^*) (Figure 2B). These freshly derived *tel1Δ rad3Δ* cells were then pooled and grown in liquid culture to obtain sufficient amount of cells at early generation to perform our biochemical analyses. Based on Southern blot analysis, we estimate that the average telomere length of *tel1Δ rad3Δ* cells utilized in our experiments is shorter than wt cells, but comparable or even slightly longer than *rad3Δ* cells (Figure 2C). Furthermore, based on amplification cycle numbers for input samples in our quantitative PCR analyses for chromatin immunoprecipitation (ChIP) assays, we can ensure that *tel1Δ rad3Δ* cells utilized in our experiments have not yet circularized their chromosomes, since primer annealing sites are completely lost after chromosome circularization [26].

**Figure 3:**
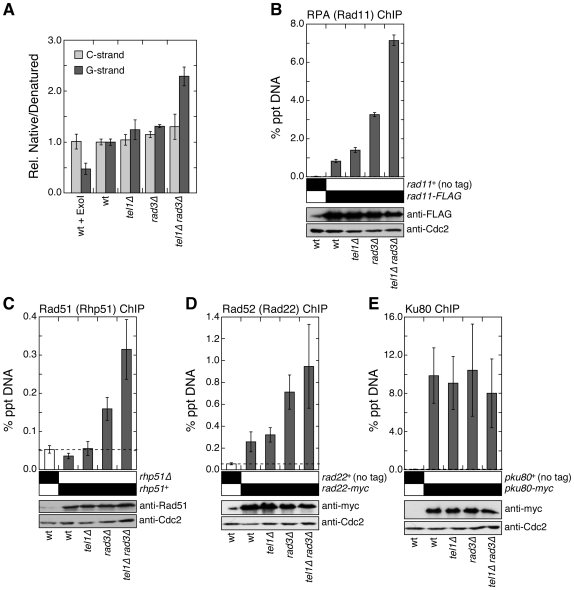
*tel1Δ rad3Δ* cells are defective in telomere protection. (A) *tel1Δ rad3Δ* cells show increase in telomeric G-tail. Genomic DNA samples from wt, *tel1Δ*, *rad3Δ*, and *tel1Δ rad3Δ* cells were prepared, and spotted onto nylon membranes to perform native dot blot analysis using strand specific probes. Hybridization signals obtained from native samples were divided by hybridization signals obtained from denatured samples, and further normalized against wt. Treatment of wt DNA with *Escherichia coli* Exo1 nuclease resulted in reduction of G-strand specific hybridization signal, but not C-strand specific signal, as expected. Mean values plus or minus one average deviation for two independently prepared genomic DNA samples are plotted. The native/denature hybridization G-strand signals were significantly higher than wt for *rad3Δ* (P = 0.032) and *tel1Δ rad3Δ* (P = 0.019), but not for *tel1Δ* (P = 0.337). For C-strand, the native/denatured hybridization signals did not differ significantly for single or double mutant cells compared to wt (P values ranged from 0.177 to 0.710). (B–E) Recruitment of Rad11^RPA^ (B), Rhp51^Rad51^ (C), Rad22^Rad52^ (D), and Ku80 (E) to telomeres in wt, *tel1Δ*, *rad3Δ*, and *tel1Δ rad3Δ* cells was monitored by quantitative ChIP assays. Protein expression levels for indicated proteins were monitored by Western blot analyses. Western blots with anti-Cdc2 were used as loading controls. For Rad11^RPA^ (B), mean values plus or minus one average deviation for two independent experiments are plotted. Compared to untagged control, Rad11^RPA^ showed significant telomere binding for wt, *tel1Δ*, *rad3Δ*, and *tel1Δ rad3Δ* (P values ranged from 0.001 to 0.022). Compared to wt cells, *rad3Δ* (P = 0.004) and *tel1Δ rad3Δ* (P = 0.003) had significant increases in RPA binding, while the increase in *tel1Δ* was not significant (P = 0.079). For Rhp51^Rad51^ (C), mean values plus or minus one standard deviation from three to five independent experiments are plotted. Compared to *rhp51Δ* cells, only *rad3Δ* (P = 0.001) and *tel1Δ rad3Δ* (P = 0.0003) showed significant binding of Rhp51^Rad51^ to telomeres. The values for *rad3Δ* and *tel1Δ rad3Δ* were also significantly different from one another (P = 0.017). For Rad22^Rad52^ (D), mean values plus or minus one standard deviation from four to six independent experiments are plotted. Compared to untagged control, Rad22^Rad52^ showed significant binding for wt, *tel1Δ*, *rad3Δ*, and *tel1Δ rad3Δ* (P values <0.0005). Compared to wt, *rad3Δ* (P = 0.001) and *tel1Δ rad3Δ* (P = 0.004) showed significant increase in Rad22^Rad52^ binding, but not *tel1Δ* (P = 0.232). The difference between *rad3Δ* and *tel1Δ rad3Δ* was not significant (P = 0.287). For Ku80 (E), mean values plus or minus one standard deviation from two to four independent experiments are plotted. Compared to untagged control, Ku80 showed significant binding in wt, *tel1Δ*, *rad3Δ*, and *tel1Δ rad3Δ* (P values <0.01), but no significant changes among wt and different mutant strains were found (P values ranged from 0.495 to 0.858).

We first examined changes in telomeric G-tail length by carrying out a series of non-denaturing native dot blot hybridization experiments using G-rich or C-rich strand specific probes for genomic DNA samples prepared from wt, *tel1Δ*, *rad3Δ* and *tel1Δ rad3Δ* cells. We found that the native hybridization signal for the probe that specifically anneals to the G-rich strand of telomeres (normalized against denatured sample), but not for the probe specific for the C-rich strand, increased significantly in *tel1Δ rad3Δ* cells (Figure 3A). Thus, we conclude that the telomeric G-tail is significantly elongated in *tel1Δ rad3Δ* cells, compared to wt, *tel1Δ*, or *rad3Δ* cells. The increase in G-tail length may be caused by a decrease in protection of the telomeric C-rich strand against degradation, or a delay in the arrival of lagging strand DNA polymerases at telomeres [17].

We next monitored recruitment of the largest subunit of RPA (replication protein A) Rad11 and the homologous recombination (HR) DNA repair proteins Rad51 and Rad52 (Rhp51 and Rad22 in fission yeast, respectively) by quantitative ChIP assays. Based on Western blot analyses, expression levels for all analyzed proteins did not change significantly, when *tel1* and/or *rad3* were deleted. We found that Rad11^RPA^, Rhp51^Rad51^, and Rad22^Rad52^ are all recruited to telomeres at significantly higher levels in *rad3Δ* and *tel1Δ rad3Δ* cells (Figure 3B–3D). While Rad22^Rad52^ recruitment to telomeres was comparable between *rad3Δ* and *tel1Δ rad3Δ* cells, Rad11^RPA^ and Rhp51^Rad51^ recruitment to telomeres was significantly higher in *tel1Δ rad3Δ* than in *rad3Δ* cells. Since *rad3Δ* cells carry much shorter telomeres than wt cells [19],[26] (Figure 2C), increased incidences of cells experiencing critically short telomeres may be responsible for increase in telomere association of RPA and HR repair factors in *rad3Δ* cells. In contrast to HR repair proteins, telomere recruitment of Ku80, involved in NHEJ repair, was not greatly affected by deletion of *tel1* and/or *rad3* (Figure 3E). The observed increase in telomere binding for RPA and Rad22^Rad52^, but not Ku, would be consistent with the notion that chromosome circularization in *tel1Δ rad3Δ* cells might occur by single strand annealing rather than NHEJ, much like in *pot1Δ* cells [29].

### Simultaneous Loss of Tel1^ATM^ and Rad3^ATR^ Leads to Defects in Recruitment of the Pot1 Sub-Complex Subunits Tpz1 and Ccq1 to Telomeres

**Figure 4:**
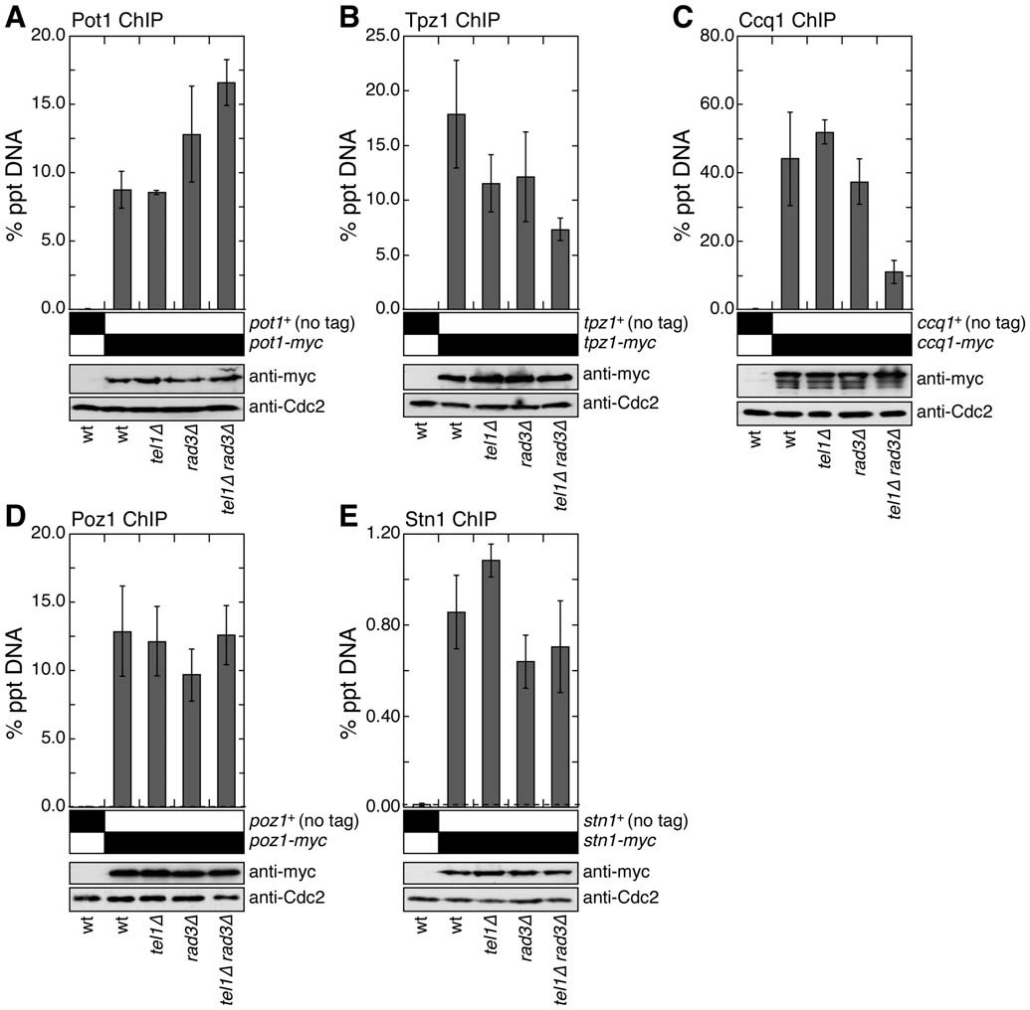
Effects of *tel1Δ rad3Δ* mutations on recruitment of telomere capping complexes. Recruitment of Pot1 (A), Tpz1 (B), Ccq1 (C), Poz1 (D), and Stn1 (E) to telomeres in wt, *tel1Δ*, *rad3Δ*, and *tel1Δ rad3Δ* cells was monitored by quantitative ChIP assays. Protein expression levels for indicated proteins were monitored by Western blot analyses. Western blots with anti-Cdc2 were used as loading controls. For Pot1 (A), mean values plus or minus one standard deviation from three to five independent experiments are plotted. Compared to untagged control, Pot1 showed significant telomere binding for wt, *tel1Δ*, *rad3Δ*, and *tel1Δ rad3Δ* (P values <0.0008). Compared to wt cells, only *tel1Δ rad3Δ* (P = 0.0005) had a significant increase in Pot1 binding, while changes in *tel1Δ* (P = 0.872) and *rad3Δ* (P = 0.147) were not significant. For Tpz1 (B), mean values plus or minus one standard deviation from three to four independent experiments are plotted. Compared to untagged control, Tpz1 showed significant telomere binding for wt, *tel1Δ*, *rad3Δ*, and *tel1Δ rad3Δ* (P values <0.002). Compared to wt cells, only *tel1Δ rad3Δ* (P = 0.006) had a significant decrease in Tpz1 binding, while decreases in *tel1Δ* (P = 0.108) and *rad3Δ* (P = 0.170) were not significant. For Ccq1 (C), mean values plus or minus one standard deviation from two to six independent experiments are plotted. Compared to untagged control, Ccq1 showed significant telomere binding for wt, *tel1Δ*, *rad3Δ*, and *tel1Δ rad3Δ* (P values <0.006). Compared to wt cells, only *tel1Δ rad3Δ* (P = 0.006) had a significant decrease in Ccq1 binding, while changes in *tel1Δ* (P = 0.337) and *rad3Δ* (P = 0.989) were not significant. For Poz1 (D), mean values plus or minus one standard deviation from four to seven independent experiments are plotted. Compared to untagged control, Poz1 showed significant telomere binding in wt, *tel1Δ*, *rad3Δ*, and *tel1Δ rad3Δ* (P values <0.0001), but no significant changes among wt and different mutant strains were found (P values ranged from 0.144 to 0.887). For Stn1 (E), mean values plus or minus one standard deviation from three to four independent experiments are plotted. Compared to untagged control, Stn1 showed significant telomere binding in wt, *tel1Δ*, *rad3Δ*, and *tel1Δ rad3Δ* (P values <0.0005), but no significant changes among wt and different mutant strains were found (P values ranged from 0.07 to 0.288).

Since we observed an increase in G-tail length and recruitment of HR repair factors in *tel1Δ rad3Δ* cells, we suspected that the integrity and/or recruitment of telomere capping complexes might be affected by the loss of Tel1^ATM^ and Rad3^ATR^. Accordingly, we monitored changes in the association of the Pot1 sub-complex (composed of Pot1, Tpz1, Poz1 and Ccq1) and the Stn1 complex (composed of Stn1 and Ten1) by quantitative ChIP assays. Previous studies have established that these complexes are likely to be independent, but both are essential for telomere protection in fission yeast [7],[15],[17],[30]. Western blot analyses indicated that expression levels for all analyzed proteins are not greatly affected by deletion of *tel1* and/or *rad3* (Figure 4).

While we did not observe any major changes in Stn1 recruitment to telomeres (Figure 4E), we observed subunit specific changes in recruitment of the Pot1 sub-complex to telomeres when Tel1^ATM^ and Rad3^ATR^ were eliminated. While Pot1 recruitment to telomeres was increased in *tel1Δ rad3Δ* cells (Figure 4A), recruitment of Tpz1 and Ccq1 was significantly reduced in *tel1Δ rad3Δ* cells (Figure 4B, 4C), and recruitment of Poz1 was not significantly affected among different genetic backgrounds (Figure 4D). Therefore, it appears that simultaneous loss of Tel1^ATM^ and Rad3^ATR^ differentially affects individual subunits of the Pot1 sub-complex. It is also worth noting that the increase in telomere association for RPA (∼9 fold) is much greater than for Pot1 (∼2 fold) in *tel1Δ rad3Δ* cells.

**Figure 5:**
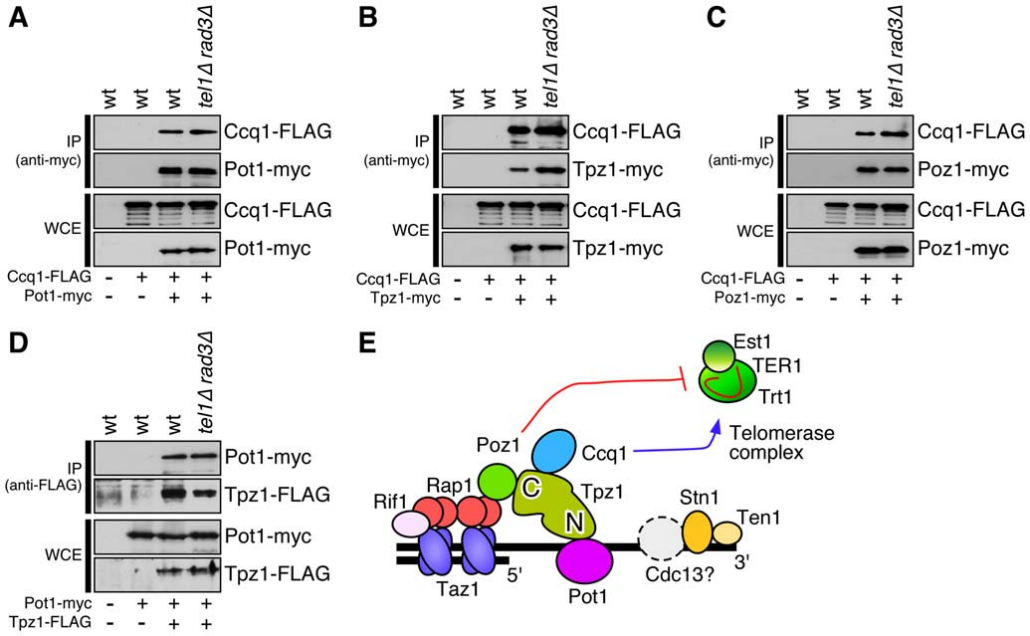
Co-IP experiments to examine the stability of the Pot1 sub-complex in wt and *tel1Δ rad3Δ*. Pairwise interactions were tested for (A) Pot1-Ccq1, (B) Tpz1-Ccq1, (C) Poz1-Ccq1, and (D) Pot1-Tpz1 in wt and *tel1Δ rad3Δ* cells. (E) A schematic representation of the proteins involved in fission yeast telomere maintenance [7],[15]. A hypothetical Cdc13-like protein that might interact with the Stn1-Ten1 complex is also shown as a dotted gray circle.

**Figure 6:**
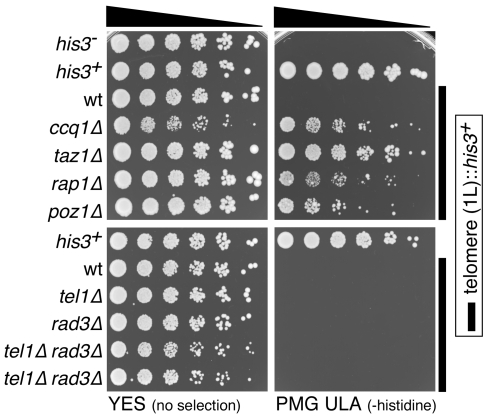
Gene silencing at telomeres is intact in *tel1Δ rad3Δ* cells. Various wt and mutant fission yeast cells that carry the *his3* ^+^ marker gene within the chromosome 1L telomere [48] were serially diluted and spotted on YES (no selection) or PMG ULA (-histidine) plates. While *ccq1Δ*, *taz1Δ*, *rap1Δ*, and *poz1Δ* disrupted telomeric silencing and allowed cells to grow on PMG ULA plates, *tel1Δ*, *rad3Δ*, or *tel1Δ rad3Δ* did not cause loss of telomeric silencing.

Given that Ccq1 and Tpz1 association to telomeres was decreased while Pot1 association was increased, we wondered if the integrity of the Pot1 sub-complex is compromised in *tel1Δ rad3Δ* cells. Therefore, we performed pairwise co-immunoprecipitation (IP) experiments among different subunits of the Pot1 sub-complex in wt and *tel1Δ rad3Δ* cells (Figure 5). Surprisingly, we did not observe any obvious changes in interactions. One possible explanation might be that asynchronous fission yeast cell cultures contain a large excess of the Pot1 sub-complex that is not bound to telomeres and thus is not regulated by Tel1/Rad3. If only the telomere-bound Pot1 sub-complex stability is affected in *tel1Δ rad3Δ* cells, co-IP assays may not be able to detect changes in complex stability. It is currently unknown if fission yeast cells contain a large pool of telomere unbound Pot1 sub-complex, but we have previously shown that telomere association of Pot1 is cell cycle regulated and occurs maximally during late S-phase [17]. Alternatively, since previous studies have demonstrated that Ccq1 can interact with the heterochromatin modulator SHREC complex [7],[31], loss of Ccq1 from telomeres might be caused by loss of interaction between SHREC and Ccq1 without affecting the stability of the telomere-bound Pot1 sub-complex. However, we found that *tel1Δ rad3Δ* cells appear to have intact heterochromatin based on the intact telomere-specific silencing of a marker gene (Figure 6). Previous studies have indicated that recruitment of Pot1 can occur independently of its N-terminal OB fold domain, required to bind G-tails at 3′ ends of telomeres, and that Rap1-Poz1 interaction can promote recruitment of the Pot1 sub-complex to the dsDNA portion of telomeres [7],[32]. In fact, based on microscopic observation [32], a majority of Pot1 may be associated with dsDNA portion of telomeric and sub-telomeric regions, and only a small fraction of the Pot1 sub-complex is bound to the extreme 3′ ends of telomeres. Therefore, we currently favor the notion that Tel1^ATM^ and Rad3^ATR^ are especially important for stabilizing the Pot1 sub-complex bound close to the 3′ ends of telomeres, but bulk of the Pot1 sub-complex, bound to the dsDNA portion of telomeres (or unbound to telomeres), are not significantly affected by simultaneous deletion of Tel1^ATM^ and Rad3^ATR^ kinases.

### Simultaneous Loss of Tel1^ATM^ and Rad3^ATR^ Leads to Defects in Recruitment of Telomerase to Telomeres

**Figure 7:**
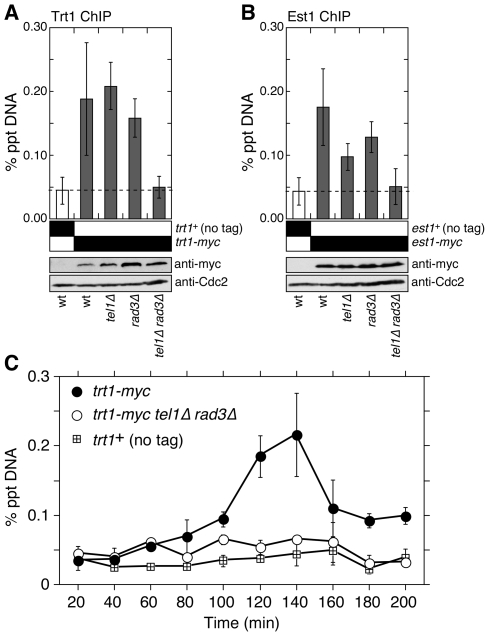
Telomerase cannot be recruited to telomeres in *tel1Δ rad3Δ* cells. (A,B) Recruitment of Trt1^TERT^ (A) and Est1 (B) to telomeres is lost in *tel1Δ rad3Δ* cells. For Trt1^TERT^ (A), mean values plus or minus one standard deviation from two to six independent experiments are plotted. Compared to untagged control, Trt1^TERT^ showed significant telomere binding in wt, *tel1Δ*, and *rad3Δ* (P values <0.004), but not in *tel1Δ rad3Δ* (P = 0.635). Compared to wt cells, only *tel1Δ rad3Δ* (P = 0.009) had significant decrease in Trt1^TERT^ binding, while changes in *tel1Δ* (P = 0.687) and *rad3Δ* (P = 0.671) were not significant. For Est1 (B), mean values plus or minus one standard deviation from two to seven independent experiments are plotted. Compared to untagged control, Est1 showed significant telomere binding in wt, *tel1Δ*, and *rad3Δ* (P values <0.002), but not in *tel1Δ rad3Δ* (P = 0.628). Compared to wt cells, *tel1Δ* (P = 0.022) and *tel1Δ rad3Δ* (P = 0.004) had significant decrease in Est1 binding, while the change in *rad3Δ* was not significant (P = 0.335). (C) The late S-phase specific recruitment of Trt1^TERT^ to telomeres is disrupted in *tel1Δ rad3Δ* cells. Cell cultures were synchronized using the temperature-sensitive *cdc25-22* allele as previously described [17], and recruitment of Trt1^TERT^ to telomeres during cell cycle was monitored by quantitative ChIP. Mean values plus or minus one average deviation from two independent experiments are plotted. Based on timing of BrdU incorporation and DNA polymerase recruitment, the S-phase occurs between 80 and 160 min [17].

Ccq1 was recently found to be important for telomerase-dependent telomere maintenance in fission yeast [7],[14]. Moreover, Ccq1 and Trt1^TERT^ can be co-immunoprecipitated, and Tpz1 pull down experiments can bring down active telomerase in a Ccq1-dependent manner. Since we found reduced association of Tpz1 and Ccq1 to telomeres in our quantitative ChIP analyses (Figure 4), we next examined if recruitment of telomerase to telomeres is affected by loss of Tel1^ATM^ and Rad3^ATR^. We found that telomere association of both the telomerase catalytic subunit Trt1^TERT^ and its regulatory subunit Est1 are significantly reduced in *tel1Δ rad3Δ* cells (Figure 7), much like in *ccq1Δ* cells [14] (Figure S1). The loss of ChIP signals were not due to loss of the telomerase complex subunits since comparable expression levels of Trt1^TERT^ and Est1 were detected by Western blots in all genetic backgrounds tested.

**Figure 8:**
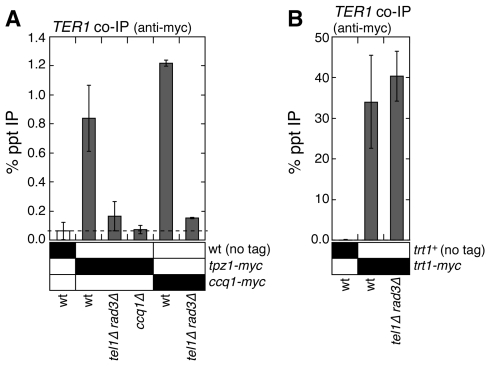
Tel1^ATM^ and Rad3^ATR^ are required for interaction between the Pot1 sub-complex and telomerase. (A) Association between the Pot1 sub-complex (Tpz1 and Ccq1) and telomerase RNA (*TER1*) is lost in *tel1Δ rad3Δ* cells. (B) Association between telomerase RNA (*TER1*) and Trt1^TERT^ is not altered in *tel1Δ rad3Δ* cells. Anti-myc IP experiments were performed, and associated *TER1* was determined by reverse transcribing associated telomerase RNA, followed by quantitative PCR. For Tpz1, mean values plus or minus one standard deviation from four to six independent experiments are plotted. Compared to untagged control, Tpz1 showed significant association with *TER1* RNA in wt (P = 0.00002), but not in *tel1Δ rad3Δ* (P = 0.115) or *ccq1Δ* (P = 0.850). For Ccq1, mean values plus or minus one standard deviation from two independent experiments are plotted. Compared to untagged control, Ccq1 showed significant association with *TER1* RNA in wt (P = 0.0000004), but not in *tel1Δ rad3Δ* (P = 0.112). For Trt1^TERT^, mean values plus or minus one standard deviation from four independent experiments are plotted. Compared to untagged control, Trt1^TERT^ showed significant association with *TER1* RNA in both wt (P = 0.0006) and *tel1Δ rad3Δ* (P = 0.0001). A difference in association between Trt1^TERT^ and *TER1* RNA in wt compared to *tel1Δ rad3Δ* was not significant (P = 0.230).

We next examined if interactions among telomerase, Tpz1 and Ccq1 are disrupted in *tel1Δ rad3Δ*. Indeed, the Ccq1-dependent interaction between the telomerase RNA subunit *TER1* and Tpz1, as well as interaction between Ccq1 and *TER1* were abolished in *tel1Δ rad3Δ* cells (Figure 8A). The loss of interaction between Tpz1-Ccq1 and telomerase is not due to disruption of the telomerase complex or degradation of telomerase RNA in *tel1Δ rad3Δ* cells, since we can pull down comparable amounts of telomerase RNA when the telomerase catalytic subunit Trt1^TERT^ was used for IP (Figure 8B). Taken together, our data indicate that the telomerase complex (Trt1-Est1-*TER1*) can no longer be recruited to telomeres in the absence of Tel1^ATM^ and Rad3^ATR^ due to the disruption of the Pot1 sub-complex and its interaction with telomerase.

## Discussion

**Figure 9:**
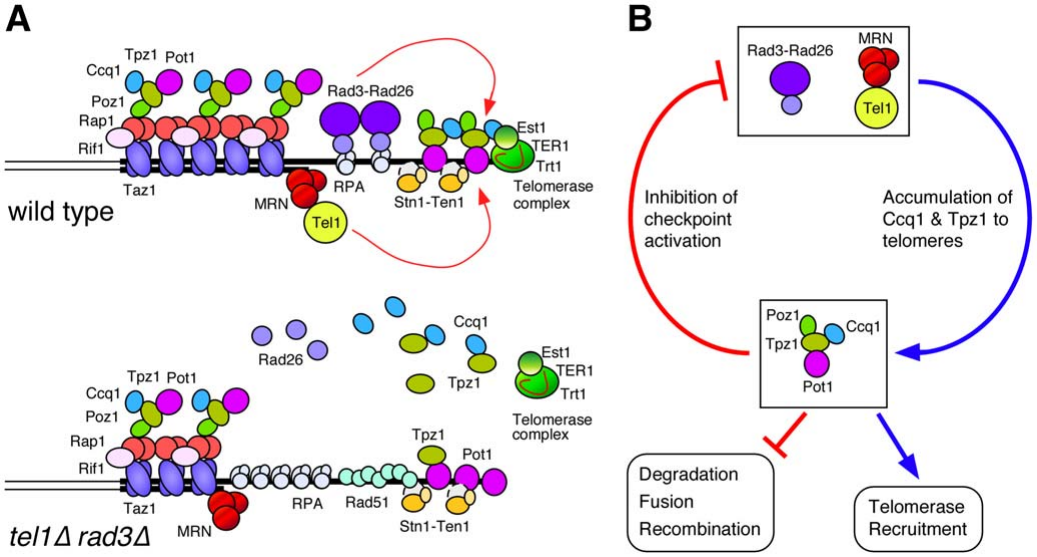
Tel1^ATM^ and Rad3^ATR^ promote telomere protection and telomerase recruitment. (A) A model for telomere dysfunction caused by simultaneous loss of Tel1^ATM^ and Rad3^ATR^ in fission yeast. The “open” or accessible state of the telomere during late S-phase, which allows recruitment of Rad3-Rad26 and telomerase, is depicted [7],[17]. For simplicity, Ku70-Ku80 and Rad22^Rad52^ are omitted from the figure. MRN (Mre11-Rad50-Nbs1) and Rad26^ATRIP^ have previously been established to function with Tel1^ATM^ and Rad3^ATR^, respectively [26],[49]. Our unpublished ChIP data indicated that MRN is still recruited to telomeres in *tel1Δ rad3Δ* cells, but Rad26^ATRIP^ is lost from telomeres in *tel1Δ rad3Δ* cells. (B) A proposed regulatory loop of Tel1^ATM^/Rad3^ATR^ and the Pot1 sub-complex, required for telomere maintenance. The Pot1 sub-complex subunit Ccq1 is involved in telomere capping and telomerase recruitment, and its recruitment is redundantly promoted by Tel1^ATM^ and Rad3^ATR^ kinases.

In this paper, we investigated the nature of telomere dysfunction caused by simultaneous deletion of the two major checkpoint kinases Tel1^ATM^ and Rad3^ATR^ in fission yeast. Results reported here support a model depicted in Figure 9A. We showed that *tel1Δ rad3Δ* cells accumulate longer G-tails (Figure 3A), suggesting possible defects in either protection against degradation of the C-rich strand or in coordination of leading and lagging strand synthesis at telomeres. The observed increases in recruitment of RPA, Rad51 and Rad52 to telomeres (Figure 3B–3D) further support the notion that *tel1Δ rad3Δ* cells are defective in protection of telomeres. Analysis of telomere complexes suggests that *tel1Δ rad3Δ* cells are defective in efficient accumulation of the shelterin subunits Tpz1 and Ccq1 (Figure 4). Moreover, we determined that *tel1Δ rad3Δ* cells were unable to recruit telomerase to telomeres due to a defect in interaction between Tpz1-Ccq1 and telomerase (Figures 7 and 8). The loss of interaction between Tpz1-Ccq1 and telomerase may be due to direct role(s) of Tel1/Rad3 in promoting this interaction, or could be indirectly caused by inefficient accumulation of Tpz1-Ccq1 at telomeres. It should also be noted that our data do not rule out the possibility that Tel1^ATM^ and Rad3^ATR^ phosphorylate different sets of substrates at telomeres.

Given that *ccq1Δ* cells were previously found to be defective in both protection of telomeres and recruitment of telomerase [7],[14], our data is consistent with the notion that all telomere defects observed in *tel1Δ rad3Δ* may primarily be caused by the failure to properly accumulate Ccq1 at telomeres. Ccq1 was also recently shown to be essential for suppressing Rad3^ATR^-dependent G_2_ checkpoint activation by telomeres [14]. Thus, it appears that fission yeast Tel1^ATM^ and Rad3^ATR^ promote accumulation of their own inhibitor Ccq1 to ensure that telomeres do not cause permanent cell cycle arrest.

The regulatory loop formed by Tel1/Rad3 and the Pot1 sub-complex (Figure 9B) ensures that telomeres that transiently become de-protected would preferentially activate Tel1/Rad3 pathways to promote recruitment of Tpz1 and Ccq1, and to re-establish proper protection of telomeres. An analogous regulatory loop appears to exist in *Drosophila*, where retrotransposons have replaced telomerase and neither the shelterin complex nor the Stn1-Ten1 complex exist, since ATM and ATR are redundantly required to promote recruitment of the telomere capping protein HOAP to telomeres [22],[23]. We suspect that mammalian ATM and ATR might also be involved in promotion of telomere capping by affecting the recruitment of the shelterin complex components.

Similar to budding yeast, where Tel1^ATM^ and Mec1^ATR^ are redundantly required to promote interaction between the G-tail binding protein Cdc13 and telomerase, our data demonstrate that fission yeast Tel1^ATM^ and Rad3^ATR^ are redundantly required to recruit telomerase to telomeres by promoting the interaction between the Pot1 sub-complex and telomerase. In budding yeast, phosphorylation of Cdc13 by Tel1^ATM^/Mec1^ATR^ kinases promotes Cdc13-Est1 interaction to facilitate telomerase recruitment [24]. Tel1^ATM^/Rad3^ATR^ kinases may also promote interaction between the Pot1 sub-complex and telomerase by phosphorylation of the Pot1 sub-complex subunits in fission yeast. Mammalian POT1-TPP1 has also been implicated in recruitment of telomerase to telomeres [6]. Thus, future studies may uncover an involvement of mammalian ATM/ATR in promoting the interaction between POT1-TPP1 and telomerase.

## Materials and Methods

### Yeast Strains and Plasmids

Fission yeast strains used in this study were constructed by standard techniques [33] and are listed in supplemental Table S1. For *tel1Δ::LEU2*, *rad3Δ::LEU2*, *pku80Δ::ura4^+^*, *taz1Δ::ura4^+^*, *rap1Δ::ura4^+^*, *rif1Δ::ura4^+^* and *rhp51Δ::ura4^+^*, original deletion strains were described previously [11], [26], [34]–[36]. For *rad11-FLAG*, *pku80-myc*, *pot1-myc*, *poz1-myc*, *stn1-myc* and *trt1-myc*, original tagged strains were described previously [17],[37],[38]. Primers listed in Table S2 were used to construct *ccq1Δ::hphMX*, *ccq1-myc*, *ccq1-FLAG*, *tpz1-myc*, *tpz1-FLAG*, *est1-myc* and *rad22-myc* by PCR-based methods [39]–[41]. The plasmids pREP41H-rad3 and pREP42-myc-rad3 were used to complement *tel1Δ rad3Δ* strains to maintain telomeres. pREP41H-rad3 carries *rad3^+^* under the control of the medium strength *nmt1* promoter and a *his3^+^* marker, while the pREP42-myc-rad3 carries *myc-rad3^+^* under the control of the medium strength *nmt1* promoter and an *ura4^+^* marker [42],[43].

### Rad3-plasmid Loss System

*tel1Δ rad3Δ* strains carrying either pREP41H-rad3 or pREP42-myc-rad3 were grown in YES liquid culture for 16 hours prior to plating onto YES plates in order to promote loss of plasmid. Small colonies were picked and simultaneously streaked on YES, YES+5 mM HU, and PMG UAL (-His) or HAL (-Ura) plates to verify the loss of *rad3^+^* and the selection marker. Several colonies that were sensitive to HU and did not grow on PMG selection plates were pooled and inoculated in YES liquid culture, and grown overnight to obtain sufficient cells for subsequent experiments.

### Southern Blot Analysis

Pulsed-field gel electrophoresis of NotI-digested chromosomal DNA was performed to monitor chromosome circularization as previously described [26]. For telomere length analysis, genomic DNA samples were digested with EcoRI, separated on a 1% agarose gel, and probed with telomere probe [44] as previously described.

### G-Tail Analysis

Native dot blot analyses were performed as described [45], with minor modifications. DNA was blotted onto Hybond-XL membrane (GE) using the BioRad Bio-Dot Microfiltration System. Hybridization was performed in Church buffer [0.25 M sodium phosphate buffer pH 7.2, 1 mM EDTA, 1% BSA, 7% SDS] at 45°C overnight with probes annealing to the G-rich strand [848: CGT GTA ACC ACG TAA CCT TGT AAC CCG ATC] or to the C-rich strand [847: GAT CGG GTT ACA AGG TTA CGT GGT TAC ACG] [46].

### ChIP Analysis

Cells were processed for ChIP and analyzed as previously described [17]. Monoclonal anti-myc (9B11; Cell Signaling) and anti-FLAG (M2-F1804; Sigma) antibodies and polyclonal anti-Rad51 antibody (A-92, Santa Cruz) were used. Percent precipitated DNA values (% ppt DNA) were calculated based on ΔCt between Input and IP samples after performing several independent triplicate SYBR Green-based real-time PCR (Bio-Rad) using telomere primers jk380 and jk381 [17].

### Co-IP of the Pot1 Sub-Complex

Cell extracts were prepared in lysis buffer [50 mM Tris pH 8.0, 150 mM NaCl, 10% glycerol, 5 mM EDTA, 0.5% NP40, 50 mM NaF, 1 mM DTT, 1 mM PMSF, 0.2 mM APMSF, 1 mM Na3VO4, ‘Complete’ protease inhibitor cocktail] using glass beads. Extracts were preincubated with 100 µg/ml Ethidium bromide for 30 min on ice. Proteins were immunoprecipitated using either monoclonal anti-myc antibody (9B11, Cell Signaling) or monoclonal anti-FLAG antibody (M2-F1804, Sigma), and Dynabeads (Invitrogen). Immunoprecipitated proteins were analyzed by Western blot analysis.

### Western Blot Analysis

Proteins in whole cell extract or from immunoprecipitations were analyzed by western blot using either monoclonal anti-FLAG antibody (M2-F1804) or monoclonal anti-myc antibody (9B11). Anti-Cdc2 antibody (y100.4, Abcam) was used for loading control.

### Co-IP of *TER1* RNA

Experiments were performed essentially as described [37]. Cell extracts were prepared in TMG100 buffer [10 mM Tris pH 8.0, 1 mM MgCl_2_, 100 mM NaCl, 10% glycerol, 1 mM EDTA, 0.1 mM DTT, 2 mM PMSF, 0.2 mM APMSF, 1 U/µl RNasin (Promega), and ‘Complete’ protease inhibitor cocktail] using glass beads. IPs were performed with 4 mg of whole cell extract in the presence of 0.5% v/v Tween20 using monoclonal anti-myc antibody (9B11) and Dynabeads (Invitrogen). Beads were subsequently washed with TMG100 buffer and treated with 0.4 mg/ml Proteinase K in [10 mM Tris pH 8.0, 100 mM NaCl, 1% SDS, 10 mM EDTA] at 37°C for 30 min. RNA was isolated using ‘Total RNA Isolation’ Kit (Clontech). RNA was reverse transcribed using M-MLV Reverse Transcriptase (Ambion) with Primer 1016 [GAT CCA TGG ATC TCA CGT AAT G], and subsequently subjected to triplicate SYBR Green-based real-time PCR analysis with primers 1015 [CAG TGT ACG TGA GTC TTC TGC CTT] and 1017 [CAA AAA TTC GTT GTG ATC TGA CAA GC]. Control reactions were also performed without reverse transcriptase to ensure that the PCR signal reflected RNA and not contaminating DNA.

### Statistical Analysis

In order to determine statistical significance of our data, two-tailed Student's t-tests were performed, and P values ≤0.05 were considered as statistically significant differences.

## Supporting Information

Figure S1 Ccq1 is required for recruitment of telomerase to telomeres. (A) Recruitment of Trt1^TERT^ and Est1 to telomeres was monitored by quantitative ChIP assays in wt and *ccq1Δ* cells. Mean values plus or minus one standard deviation from two to six independent experiments are plotted. Compared to untagged control, Trt1^TERT^ showed significant telomere binding in *ccq1^+^* (P = 0.002), but not in *ccq1Δ* (P = 0.052). Compared to untagged control, Est1 showed significant telomere binding in *ccq1^+^* (P = 0.000002), but not in *ccq1Δ* (P = 0.143). (B) Protein expression levels for Trt1^TERT^ (top) and Est1 (bottom) were monitored by Western blot analyses. Western blots with anti-Cdc2 were used as loading controls.
(0.17 MB TIF)

Table S1 Fission yeast strains used in this study.
(0.20 MB DOC)

Table S2 DNA primers used in strain construction.
(0.06 MB DOC)
