# Supplementary figures and images for: Fission Yeast Tel1ATM and Rad3ATR Promote Telomere Protection and Telomerase Recruitment
Source: PLoS Genet. 2009 Aug 28;5(8):e1000622. doi: 10.1371/journal.pgen.1000622 (PMC2726628; doi:10.1371/journal.pgen.1000622)

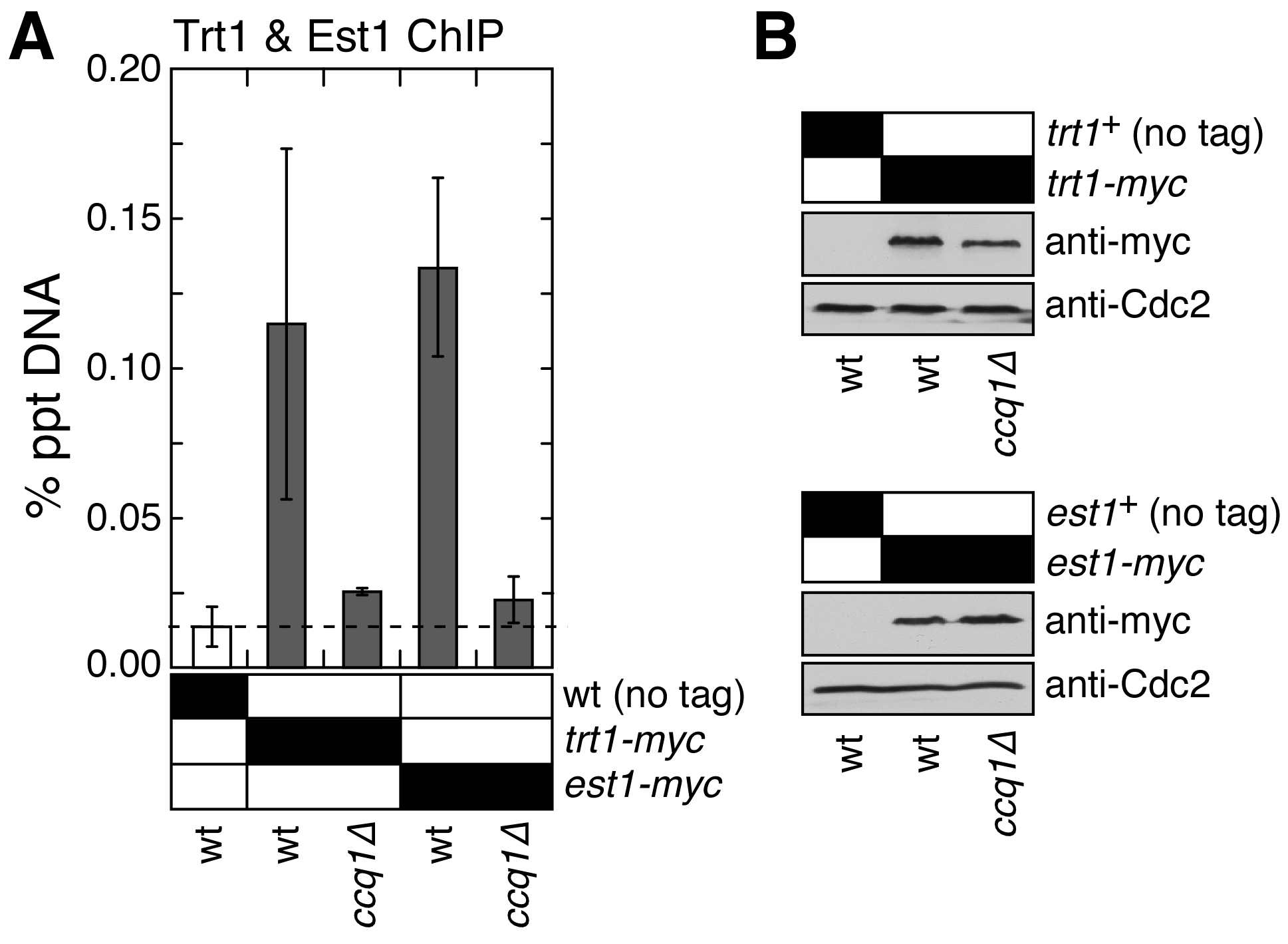

Supplement: Figure S1 — Ccq1 is required for recruitment of telomerase to telomeres. (A) Recruitment of Trt1TERT and Est1 to telomeres was monitored by quantitative ChIP assays in wt and ccq1Δ cells. Mean values plus or minus one standard deviation from two to six independent experiments are plotted. Compared to untagged control, Trt1TERT showed significant telomere binding in ccq1+ (P = 0.002), but not in ccq1Δ (P = 0.052). Compared to untagged control, Est1 showed significant telomere binding in ccq1+ (P = 0.000002), but not in ccq1Δ (P = 0.143). (B) Protein expression levels for Trt1TERT (top) and Est1 (bottom) were monitored by Western blot analyses. Western blots with anti-Cdc2 were used as loading controls. (0.17 MB TIF) [file pgen.1000622.s001.tif]
