# Supplementary material for: Fission Yeast Tel1ATM and Rad3ATR Promote Telomere Protection and Telomerase Recruitment
Source: PLoS Genet. 2009 Aug 28;5(8):e1000622. doi: 10.1371/journal.pgen.1000622 (PMC2726628; doi:10.1371/journal.pgen.1000622)
Supplement: Table S1 — Fission yeast strains used in this study. (0.20 MB DOC) [file pgen.1000622.s002.doc]

| **Supplemental Table S1.** Fission yeast strains used in this study. | | | |
| --- | --- | --- | --- |
| **Figure** | | **Strain** | **Full Genotype** |
| 1A | wt | TN2411 | *h^-^ leu1-32 ura4-D18 his3-D1* |
|  | *tel1Δ rad3Δ* | TN1761 | *h^-^ leu1-32 ura4-D18 ade6-M210 his3-D1 tel1Δ::LEU2 rad3Δ::LEU2* |
|  | *tel1Δ rad3Δ pku80Δ* | LS4873 | *h^-^ leu1-32 ura4-D18 ade6-M210 his3-D1 tel1Δ::LEU2 rad3Δ::LEU2 pku80Δ::ura4^+^* |
|  | *tel1Δ rad3Δ taz1Δ* | LS4797 | *h^-^ leu1-32 ura4-D18 ade6-M210 his3-D1 tel1Δ::LEU2 rad3Δ::LEU2 taz1Δ::ura4^+^* |
|  | *tel1Δ rad3Δ rap1Δ* | LS4731 | *h^-^ leu1-32 ura4-D18 ade6-M210 his3-D1 tel1Δ::LEU2 rad3Δ::LEU2 rap1Δ::ura4^+^* |
|  | *tel1Δ rad3Δ rif1Δ* | LS4878 | *h^-^ leu1-32 ura4-D18 ade6-M210 his3-D1 tel1Δ::LEU2 rad3Δ::LEU2 rif1Δ::ura4^+^* |
|  |  |  |  |
| 2 | wt | CF199 | *h^-^ leu1-32 ura4-D18 ade6-M210 his3-D1* |
|  | *tel1Δ* | TN1343 | *h^-^ leu1-32 ura4-D18 ade6-M210 his3-D1 tel1Δ::LEU2* |
|  | *rad3Δ* | TN1374 | *h^-^ leu1-32 ura4-D18 ade6-M210 his3-D1 rad3Δ::LEU2* |
|  | *tel1Δ rad3Δ* + rad3 pld | TN4328^a^ | *h^-^ leu1-32 ura4-D18 ade6-M210 his3-D1 tel1Δ::LEU2 rad3Δ::LEU2 //pREP41H-rad3* |
|  |  |  |  |
| 3A | wt | CF199 | *h^-^ leu1-32 ura4-D18 ade6-M210 his3-D1* |
|  | *tel1Δ* | TN1343 | *h^-^ leu1-32 ura4-D18 ade6-M210 his3-D1 tel1Δ::LEU2* |
|  | *rad3Δ* | TN1374 | *h^-^ leu1-32 ura4-D18 ade6-M210 his3-D1 rad3Δ::LEU2* |
|  | *tel1Δ rad3Δ* | TN4328^a^ | *h^-^ leu1-32 ura4-D18 ade6-M210 his3-D1 tel1Δ::LEU2 rad3Δ::LEU2 //pREP41H-rad3* |
|  |  |  |  |
| 3B | wt | TN2411 | *h^-^ leu1-32 ura4-D18 his3-D1* |
|  | *rad11-FLAG* | TN5599 | *h^-^ leu1-32 ura4-D18 his3-D1 rad11^+^-5FLAG::kanMX* |
|  | *rad11-FLAG tel1Δ* | TN6299 | *h^-^ leu1-32 ura4-D18 ade6-M210 his3-D1 tel1Δ::LEU2 rad11^+^-5FLAG::kanMX* |
|  | *rad11-FLAG rad3Δ* | TN6295 | *h^-^ leu1-32 ura4-D18 his3-D1 rad3Δ::LEU2 rad11^+^-5FLAG::kanMX* |
|  | *rad11-FLAG tel1Δ rad3Δ* | TN8069^a^ | *h^-^ leu1-32 ura4-D18 ade6-M210 his3-D1 tel1Δ::LEU2 rad3Δ::LEU2 rad11^+^-5FLAG::kanMX //pREP41H-rad3* |
|  |  |  |  |
| 3C | *rhp51Δ* | CF436 | *h^+^ leu1-32 ura4-D18 ade6-M216 his3-D1 rhp51Δ::ura4^+^* |
|  | wt | TN2411 | *h^-^ leu1-32 ura4-D18 his3-D1* |
|  | *tel1Δ* | TN1343 | *h^-^ leu1-32 ura4-D18 ade6-M210 his3-D1 tel1Δ::LEU2* |
|  | *rad3Δ* | TN1373 | *h^+^ leu1-32 ura4-D18 ade6-M210 his3-D1 rad3Δ::LEU2* |
|  | *tel1Δ rad3Δ* | TN4328^a^ | *h^-^ leu1-32 ura4-D18 ade6-M210 his3-D1 tel1Δ::LEU2 rad3Δ::LEU2 //pREP41H-rad3* |
|  |  |  |  |
| 3D | wt | TN2411 | *h^-^ leu1-32 ura4-D18 his3-D1* |
|  | *rad22-myc* | LK6713 | *h^+^ leu1-32 ura4-D18 his3-D1 rad22^+^-13myc::kanMX6* |
|  | *rad22-myc tel1Δ* | LK7598 | *h^-^ leu1-32 ura4-D18 ade6-M210 his3-D1 tel1Δ::LEU2 rad22^+^-13myc::kanMX6* |
|  | *rad22-myc rad3Δ* | LK8184 | *h^-^ leu1-32 ura4-D18 ade6-M210 his3-D1 rad3Δ::LEU2 rad22^+^-13myc::kanMX6* |
|  | *rad22-myc tel1Δ rad3Δ* | TN8080^a^ | *h^-^ leu1-32 ura4-D18 ade6-M210 his3-D1 tel1Δ::LEU2 rad3Δ::LEU2 rad22^+^-13myc::kanMX6 //pREP41H-rad3* |
|  |  |  |  |
| 3E | wt | TN2411 | *h^-^ leu1-32 ura4-D18 his3-D1* |
|  | *pku80-myc* | TN7668 | *h^-^ leu1-32 ura4-D18 ade6-M216 his3-D1 pku80^+^-G_8_-13myc::kanMX6* |
|  | *pku80-myc tel1Δ* | TN7810 | *h^-^ leu1-32 ura4-D18 ade6-M210 his3-D1 tel1Δ::LEU2 pku80^+^-G_8_-13myc::kanMX6* |
|  | *pku80-myc rad3Δ* | TN7805 | *h^-^ leu1-32 ura4-D18 ade6-M210 his3-D1 rad3Δ::LEU2 pku80^+^-G_8_-13myc::kanMX6* |
|  | *pku80-myc tel1Δ rad3Δ* | TN8084^a^ | *h^-^ leu1-32 ura4-D18 ade6-M210 his3-D1 tel1Δ::LEU2 rad3Δ::LEU2 pku80^+^-G_8_-13myc::kanMX6 //pREP41H-rad3* |
|  |  |  |  |
| 4A | wt | TN2411 | *h^-^ leu1-32 ura4-D18 his3-D1* |
|  | *pot1-myc* | BAM4291 | *h^-^ leu1-32 ura4-D18 his3-D1 pot1^+^-13myc::kanMX6* |
|  | *pot1-myc tel1Δ* | TN6174 | *h^-^ leu1-32 ura4-D18 ade6-M210 his3-D1 tel1Δ::LEU2 pot1^+^-13myc::kanMX6* |
|  | *pot1-myc rad3Δ* | TN6161 | *h^-^ leu1-32 ura4-D18 his3-D1 rad3Δ::LEU2 pot1^+^-13myc::kanMX6* |
|  | *pot1-myc tel1Δ rad3Δ* | TN6553^a^ | *h^+^ leu1-32 ura4-D18 ade6-M210 his3-D1 tel1Δ::LEU2 rad3Δ::LEU2 pot1^+^-13myc::kanMX6 //pREP41H-rad3* |
|  |  |  |  |
| 4B | wt | TN2411 | *h^-^ leu1-32 ura4-D18 his3-D1* |
|  | *tpz1-myc* | TN7196 | *h^-^ leu1-32 ura4-D18 his3-D1 tpz1^+^-13myc::kanMX6* |
|  | *tpz1-myc tel1Δ* | TN8521 | *h^-^ leu1-32 ura4-D18 ade6-M210 his3-D1 tel1Δ::LEU2 tpz1^+^-13myc::kanMX6* |
|  | *tpz1-myc rad3Δ* | TN8526 | *h^-^ leu1-32 ura4-D18 his3-D1 rad3Δ::LEU2 tpz1^+^-13myc::kanMX6* |
|  | *tpz1-myc tel1Δ rad3Δ* | TN8442^a^ | *h^-^ leu1-32 ura4-D18 ade6-M210 his3-D1 tel1Δ::LEU2 rad3Δ::LEU2 tpz1^+^-13myc::kanMX6 //pREP41H-rad3* |
|  |  |  |  |
| 4C | wt | TN2411 | *h^-^ leu1-32 ura4-D18 his3-D1* |
|  | *ccq1-myc* | TN7217 | *h^-^ leu1-32 ura4-D18 his3-D1 ccq1^+^-13myc::kanMX6* |
|  | *ccq1-myc tel1Δ* | TN6174 | *h^-^ leu1-32 ura4-D18 ade6-M210 his3-D1 tel1Δ::LEU2 ccq1^+^-13myc::kanMX6* |
|  | *ccq1-myc rad3Δ* | TN8519 | *h^-^ leu1-32 ura4-D18 his3-D1 rad3Δ::LEU2 ccq1^+^-13myc::kanMX6* |
|  | *ccq1-myc tel1Δ rad3Δ* | TN8439^a^ | *h^-^ leu1-32 ura4-D18 ade6-M210 his3-D1 tel1Δ::LEU2 rad3Δ::LEU2 ccq1^+^-13myc-::kanMX6 //pREP41H-rad3* |
|  |  |  |  |
| 4D | wt | TN2411 | *h^-^ leu1-32 ura4-D18 his3-D1* |
|  | *poz1-myc* | YTC6682 | *h^-^ leu1-32 ura4-D18 his3-D1 poz1^+^-13myc::kanMX6* |
|  | *poz1-myc tel1Δ* | TN8510 | *h^-^ leu1-32 ura4-D18 ade6-M210 his3-D1 tel1Δ::LEU2 poz1^+^-13myc::kanMX6* |
|  | *poz1-myc rad3Δ* | TN8514 | *h^-^ leu1-32 ura4-D18 his3-D1 rad3Δ::LEU2 poz1^+^-13myc::kanMX6* |
|  | *poz1-myc tel1Δ rad3Δ* | TN8435^a^ | *h^-^ leu1-32 ura4-D18 ade6-M210 his3-D1 tel1Δ::LEU2 rad3Δ::LEU2 poz1^+^-13myc::kanMX6 //pREP41H-rad3* |
|  |  |  |  |
| 4E | wt | TN2411 | *h^-^ leu1-32 ura4-D18 his3-D1* |
|  | *stn1-myc* | YTC6733 | *h^-^ leu1-32 ura4-D18 his3-D1 stn1^+^-13myc::kanMX6* |
|  | *stn1-myc tel1Δ* | TN8529 | *h^-^ leu1-32 ura4-D18 ade6-M210 his3-D1 tel1Δ::LEU2 stn1^+^-13myc::kanMX6* |
|  | *stn1-myc rad3Δ* | TN8533 | *h^-^ leu1-32 ura4-D18 his3-D1 rad3Δ::LEU2 stn1^+^-13myc::kanMX6* |
|  | *stn1-myc tel1Δ rad3Δ* | TN8447^a^ | *h^-^ leu1-32 ura4-D18 ade6-M210 his3-D1 tel1Δ::LEU2 rad3Δ::LEU2 stn1^+^-13myc::kanMX6 //pREP41H-rad3* |
|  |  |  |  |
| 5A | wt | TN2411 | *h^-^ leu1-32 ura4-D18 his3-D1* |
|  | *ccq1-FLAG* | YTC6732 | *h^-^ leu1-32 ura4-D18 his3-D1 ccq1^+^-5FLAG::kanMX6* |
|  | *ccq1-FLAG pot1-myc* | TN6864 | *h^-^ leu1-32 ura4-D18 his3-D1 pot1^+^-13myc::kanMX6 ccq1^+^-5FLAG::kanMX6* |
|  | *ccq1-FLAG pot1-myc tel1Δ rad3Δ* | BAM9776^a^ | *h^+^ leu1-32 ura4-D18 ade6-M210 his3-D1 tel1Δ::LEU2 rad3Δ::LEU2 pot1^+^-13myc::kanMX6 ccq1^+^-5FLAG::kanMX6 //pREP41H-rad3* |
|  |  |  |  |
| 5B | wt | TN2411 | *h^-^ leu1-32 ura4-D18 his3-D1* |
|  | *ccq1-FLAG* | YTC6732 | *h^-^ leu1-32 ura4-D18 his3-D1 ccq1^+^-5FLAG::kanMX6* |
|  | *ccq1-FLAG tpz1-myc* | TN7506 | *h^-^ leu1-32 ura4-D18 his3-D1 tpz1^+^-13myc::kanMX6 ccq1^+^-5FLAG::kanMX6* |
|  | *ccq1-FLAG tpz1-myc tel1Δ rad3Δ* | BAM9775^a^ | *h^+^ leu1-32 ura4-D18 ade6-M210 his3-D1 tel1Δ::LEU2 rad3Δ::LEU2 tpz1^+^-13myc::kanMX6 ccq1^+^-5FLAG::kanMX6 //pREP41H-rad3* |
|  |  |  |  |
| 5C | wt | TN2411 | *h^-^ leu1-32 ura4-D18 his3-D1* |
|  | *ccq1-FLAG* | YTC6732 | *h^-^ leu1-32 ura4-D18 his3-D1 ccq1^+^-5FLAG::kanMX6* |
|  | *ccq1-FLAG poz1-myc* | TN6935 | *h^-^ leu1-32 ura4-D18 his3-D1 poz1^+^-13myc::kanMX6 ccq1^+^-5FLAG::kanMX6* |
|  | *ccq1-FLAG poz1-myc tel1Δ rad3Δ* | BAM9773^a^ | *h^-^ leu1-32 ura4-D18 ade6-M210 his3-D1 tel1Δ::LEU2 rad3Δ::LEU2 poz1^+^-13myc::kanMX6 ccq1^+^-5FLAG::kanMX6 //pREP41H-rad3* |
|  |  |  |  |
| 5D | wt | TN2411 | *h^-^ leu1-32 ura4-D18 his3-D1* |
|  | *pot1-myc* | BAM4291 | *h^-^ leu1-32 ura4-D18 his3-D1 pot1^+^-13myc::kanMX6* |
|  | *pot1-myc tpz1-FLAG* | YTC7626 | *h^-^ leu1-32 ura4-D18 his3-D1 pot1^+^-13myc::kanMX6 tpz1^+^-5FLAG-TEV-AviTag::kanMX6* |
|  | *pot1-myc tpz1-FLAG tel1Δ rad3Δ* | BAM9427^a^ | *h^-^ leu1-32 ura4-D18 ade6-M210 his3-D1 tel1Δ::LEU2 rad3Δ::LEU2 pot1^+^-13myc::kanMX6 tpz1^+^-5FLAG-TEV-AviTag::kanMX6 //pREP41H-rad3* |
|  |  |  |  |
| 6 | *his^-^* | TN2411 | *h^-^ leu1-32 ura4-D18 his3-D1* |
|  | *his^+^* | TN3784 | *h^-^ leu1-32 ura4-D18* |
|  | *telomere (1L)::his3^+^* | CF52 | *h^90^ leu1-32 ura4-DS/E ade6-M210 his3-D1 his3^+^::tel(1L)* |
|  | *telomere (1L)::his3^+^ ccq1Δ* | TN9133 | *h^-^ leu1-32 ura4-D18 his3-D1 ccq1Δ::hphMX his3^+^::tel(1L)* |
|  | *telomere (1L)::his3^+^ taz1Δ* | CF61 | *h^90^ leu1-32 ura4-DS/E ade6-M210 his3-D1 taz1Δ::ura4^+^ his3^+^:tel(1L)* |
|  | *telomere (1L)::his3^+^ rap1* | TN9136 | *h^-^ leu1-32 ura4-D18 his3-D1 rap1Δ::ura4^+^ his3^+^::tel(1L)* |
|  | *telomere (1L)::his3^+^ poz1* | TN9135 | *h^90^ leu1-32 ura4-D18 ade6-M216 his3-D1 poz1Δ::kanMX6 his3^+^::tel(1L)* |
|  | *telomere (1L)::his3^+^ tel1* | TN9127 | *h^-^ leu1-32 ura4-DS/E ade6-M210 his3-D1 tel1Δ::LEU2 his3^+^::tel(1L)* |
|  | *telomere (1L)::his3^+^ rad3Δ* | TN9129 | *h^-^ leu1-32 ura4-DS/E ade6-M210 his3-D1 rad3Δ::LEU2 his3^+^::tel(1L)* |
|  | *telomere (1L)::his3^+^ tel1Δ rad3Δ* | TN9153^a^ | *h^-^ leu1-32 ura4-DS/E or ura4-D18 ade6-M210 his3-D1 tel1Δ::LEU2 rad3Δ::LEU2 his3^+^::tel(1L) //pREP42-myc-rad3* |
|  |  |  |  |
| 7A | wt | TN2411 | *h^-^ leu1-32 ura4-D18 his3-D1* |
|  | *trt1-myc* | TN7706 | *h^-^ leu1-32 ura4-D18 his3-D1 trt1^+^-G_8_-13myc::kanMX6* |
|  | *trt1-myc tel1Δ* | TN7761 | *h^-^ leu1-32 ura4-D18 ade6-M216 his3-D1 tel1Δ::LEU2 trt1^+^-G_8_-13myc::kanMX6* |
|  | *trt1-myc rad3Δ* | TN7767 | *h^-^ leu1-32 ura4-D18 ade6-M216 his3-D1 rad3Δ::LEU2 trt1^+^-G_8_-13myc::kanMX6* |
|  | *trt1-myc tel1Δ rad3Δ* | TN8054^a^ | *h^-^ leu1-32 ura4-D18 ade6-M210 his3-D1 tel1Δ::LEU2 rad3Δ::LEU2 trt1^+^-G_8_-13myc::kanMX6 //pREP41H-rad3* |
|  |  |  |  |
| 7B | wt | TN2411 | *h^-^ leu1-32 ura4-D18 his3-D1* |
|  | *est1-myc* | BAM5087 | *h^-^ leu1-32 ura4-D18 his3-D1 est1^+^-13myc::kanMX6* |
|  | *est1-myc tel1Δ* | TN7796 | *h^-^ leu1-32 ura4-D18 ade6-M210 his3-D1 tel1Δ::LEU2 est1^+^-13myc::kanMX6* |
|  | *est1-myc rad3Δ* | TN7791 | *h^-^ leu1-32 ura4-D18 his3-D1 rad3Δ::LEU2 est1^+^-13myc::kanMX6* |
|  | *est1-myc tel1Δ rad3Δ* | TN8058^a^ | *h^-^ leu1-32 ura4-D18 ade6-M210 his3-D1 tel1Δ::LEU2 rad3Δ::LEU2 est1^+^-13myc::kanMX6 //pREP41H-rad3* |
|  |  |  |  |
| 7C | *trt1^+^* (no tag) | SS5264 | *h^-^ leu1-32 ura4-D18 his3-D1 cdc25-22* |
|  | *trt1-myc* | TN7708 | *h^-^ leu1-32 ura4-D18 his3-D1 trt1^+^-G_8_-13myc::kanMX6 cdc25-22* |
|  | *trt1-myc tel1Δ rad3Δ* | TN8197^a^ | *h^-^ leu1-32 ura4-D18 ade6-M210 his3-D1 trt1^+^-G_8_-13myc::kanMX6 tel1::LEU2 rad3::LEU2 cdc25-22 //pREP41H-rad3* |
|  |  |  |  |
| 8A | wt | TN2411 | *h^-^ leu1-32 ura4-D18 his3-D1* |
|  | *tpz1-myc* | TN7196 | *h^-^ leu1-32 ura4-D18 his3-D1 tpz1^+^-13myc::kanMX6* |
|  | *tpz1-myc tel1Δ rad3Δ* | TN8442 | *h^-^ leu1-32 ura4-D18 ade6-M210 his3-D1 tel1Δ::LEU2 rad3Δ::LEU2 tpz1^+^-13myc::kanMX6 //pREP41H-rad3* |
|  | *tpz1-myc ccq1Δ* | TN9011 | *h^-^ leu1-32 ura4-D18 his3-D1 ccq1Δ::hphMX tpz1^+^-13myc::kanMX6* |
|  | *ccq1-myc* | TN7217 | *h^-^ leu1-32 ura4-D18 his3-D1 ccq1^+^-13myc::kanMX6* |
|  | *ccq1-myc tel1Δ rad3Δ* | TN8439^a^ | *h^-^ leu1-32 ura4-D18 ade6-M210 his3-D1 tel1Δ::LEU2 rad3Δ::LEU2 ccq1^+^-13myc::kanMX6 //pREP41H-rad3* |
|  |  |  |  |
| 8B | wt | TN2411 | *h^-^ leu1-32 ura4-D18 his3-D1* |
|  | *trt1-myc* | TN7706 | *h^-^ leu1-32 ura4-D18 his3-D1 trt1^+^-G_8_-13myc::kanMX6* |
|  | *trt1-myc tel1Δ rad3Δ* | TN8054^a^ | *h^-^ leu1-32 ura4-D18 ade6-M210 his3-D1 tel1Δ::LEU2 rad3Δ::LEU2 trt1^+^-G_8_-13myc::KanMX6 //pREP41H-rad3* |
|  |  |  |  |
| S1 | wt | TN2411 | *h^-^ leu1-32 ura4-D18 his3-D1* |
|  | *trt1-myc* | TN7706 | *h^-^ leu1-32 ura4-D18 his3-D1 trt1^+^-G_8_-13myc::kanMX6* |
|  | *trt1-myc ccq1Δ* | TN8963 | *h^-^ leu1-32 ura4-D18 his3-D1 ccq1Δ::hphMX trt1^+^-G_8_-13myc::kanMX6* |
|  | *est1-myc* | BAM5087 | *h^-^ leu1-32 ura4-D18 his3-D1 est1^+^-13myc::kanMX6* |
|  | *est1-myc ccq1Δ* | TN8994 | *h^-^ leu1-32 ura4-D18 his3-D1 ccq1Δ::hphMX est1^+^-13myc::kanMX6* |

^a^Strains that have lost the Rad3 plasmid (*pREP41H-rad3* or *pREP42-myc-rad3*) were used in experiments.
