# Supplementary material for: Fission Yeast Tel1ATM and Rad3ATR Promote Telomere Protection and Telomerase Recruitment
Source: PLoS Genet. 2009 Aug 28;5(8):e1000622. doi: 10.1371/journal.pgen.1000622 (PMC2726628; doi:10.1371/journal.pgen.1000622)
Supplement: Table S2 — DNA primers used in strain construction. (0.06 MB DOC) [file pgen.1000622.s003.doc]

| **Supplemental Table S2.** DNA primers used in strain construction. | | |
| --- | --- | --- |
| **Strain** | **Primer Name** | **Primer Sequence (5’ to 3’)** |
| *ccq1Δ::hphMX* | ccq1-T8 | aacaacatttgggtggttatttgta |
|  | ccq1-KO(x)^a^ | ggggatccgtcgacctgcagcgtacgaCCTCGTATTTCGTATCACTTTCATTG |
|  | ccq1-KO/tag(y)^a^ | gtttaaacgagctcgaattcatcgatGAACTTCAGTGGAATCAACTATAAAAAGTTC |
|  | ccq1-B6 | tgttgatattgcatcttttgaggatgcatat |
|  |  |  |
| *ccq1-myc* and *ccq1-FLAG* | ccq1-tagT^a^ | AGACCTTAAAAATTTTAGAGCAGAAGTCTCTTCCCAAATTTACTCCACATAATCAATCACCAAGGATTATTGATTCTAACCGGATCCCCGGGTTAATTAA |
|  | ccq1-KO2/tagB^a^ | GACATAATTTAATCAAATAACCGTATGACTAAACGAACTTTTTATAGTTGATTCCACTGAAGTTCTTGATCGTTAATGATGAATTCGAGCTCGTTTAAAC |
|  |  |  |
| *tpz1-myc* and *tpz1-FLAG* | tpz1-T12 | TGCAATGAGCAGATAGAGCTTGAATAC |
|  | tpz1-tag(x)^a^ | ggggatccgtcgacctgcagcgtacgaGCTTTTGTTTCGAAACTCCTCTATTTTTTTCC |
|  | tpz1-tag/KO(y)^a^ | gtttaaacgagctcgaattcatcgatTGCTGTATCACATATCTTCCTTTTTTACTAACA |
|  | tpz1-B9 | ATTGCTTTCCACATACTGCCTT |
|  |  |  |
| *est1-myc* | est1-T1 | ATTGAGTTATCATTTCAGAATTTGTG |
|  | est1-B1(x)^a^ | ggggatccgtcgacctgcagcgtacgaGGAAAGCAATAAATTTAGTA |
|  | est1-T2(y)^a^ | gtttaaacgagctcgaattcatcgatGATGCTTTGAATTAACAGAG |
|  | est1-B2 | CTAATGGGCACGCTATTCCTATAGC |
|  |  |  |
| *rad22-myc* | rad22-tagT^a^ | GAACAAATTCTGATCCTCAGTCGGCAATGAGGTCGCGAGAAAACTACGATGCTACGGTGGATAAGAAAGCCAAAAAAGGACGGATCCCCGGGTTAATTAA |
|  | rad22-tagB^a^ | TATTGGCGGAAAGAGGATCAAGGTGGCAGGCAGCCTTTTGCCGGATAGCAATTGAATATTCAGACAATTATTACATAGATGAATTCGAGCTCGTTTAAAC |

^a^Underlined sequences anneal to *kanMX6*/*hphMX6*.
